# Nature experience from yards provide an important space for mental health during Covid-19

**DOI:** 10.1038/s42949-023-00094-0

**Published:** 2023-03-10

**Authors:** Brenda B. Lin, Chia-chen Chang, Thomas Astell-Burt, Xiaoqi Feng, John Gardner, Erik Andersson

**Affiliations:** 1 CSIRO Land & Water, GPO Box 2583, Brisbane, QLD 4001 Australia; 2 Department of Evolution and Ecology, University of California, Davis, CA USA; 3 School of Health and Society, Faculty of Arts, Humanities and Social Sciences, University of Wollongong, Wollongong, NSW Australia; 4 Population Wellbeing and Environment Research Lab (PowerLab), Sydney, NSW Australia; 5 School of Population Health, Faculty of Medicine and Health, University of New South Wales, Sydney, NSW Australia; 6 The George Institute for Global Health, Sydney, NSW Australia; 7 Stockholm Resilience Centre, Stockholm University, Stockholm, Sweden; 8 Ecosystems and Environment Research Program, University of Helsinki, Helsinki, Finland; 9 Research Unit for Environmental Sciences and Management, North-West University, Potchefstroom, South Africa

**Keywords:** Environmental impact, Geography, Psychology and behaviour

## Abstract

Urban dwellers’ use of public and private green spaces may have changed during the early years of the Covid-19 pandemic due to movement restriction. A survey was deployed in Brisbane and Sydney, Australia 1 year after the start of Covid-19 restrictions (April 2021) to explore relationships of mental health and wellbeing to different patterns of private yard versus public green space visitation. More frequent yard use during the initial year of Covid-19 was correlated with lower stress, depression, and anxiety and higher wellbeing. However, greater duration of yard visits (week prior to survey) was associated with higher stress, anxiety, and depression scores, potentially because individuals may seek to use nature spaces immediately available for emotional regulation during difficult times. The results highlight the importance of yards for mental health and wellbeing during the Covid-19 pandemic and that relationships between nature interaction and mental health may be context and timeframe dependent.

## Introduction

Several decades of research has investigated the role of nature experiences on benefits for human health and well-being^1–5^, with research showing consistent findings across cultures and age groups^6–8^. The recognition of the importance of nature interaction for health and wellbeing has led to a breadth of research to better understand how different engagement or design of nature spaces contribute to the benefits gained^9–12^. Public health studies have shown that people who live or move closer to greener urban areas benefit from sustained improvements in their mental health^13–15^. Increasingly, there is a desire to understand how different pathways of nature interaction leads to greater mental health and well-being benefits, especially at a time when mental health is a pervasive health issue, and people are seeking treatments^16–18^.

One particular area of focus of this research areas has developed regarding cities and their provision of nature for improved health outcomes, especially in areas where increasing densification has led to the gradual loss of both public and private green spaces available for urban communities^19,20^. Green spaces in cities contribute to environmental benefits that impact on health (e.g. cleaner air, stormwater capture), and they can provide spaces and opportunities for actively pursuing multiple wellbeing ecosystem services, including social and recreational activities, physical exercise, as well as nature connection—all of which can impact on mental health and wellbeing of an individual^11,17,21^. Thus, urban green spaces can provide many opportunities for urban dwellers to gain many health benefits if they have the orientation and desire to engage with them^22^.

The question of how green spaces are distributed and accessed has come into stark contrast during the Covid-19 pandemic^23–25^. Much recent literature has highlighted the importance of urban green space during Covid-19 as many residents across cities globally experienced lockdowns, including working from home, online learning, and travel limits. While some urban dwellers increased their use of public green space, many also reduced their use because of access restrictions to public green spaces as well as concerns around the lack of social-distancing or overcrowding, especially for females and older residents^24,26,27^. People also changed the way they used green space to meet their needs during that time with social isolation reducing the extent, type and distance of green spaces visited and others deciding to use private green spaces in lieu of public green space to stay socially-distanced^28,29^. On the other hand, some individuals began using urban green spaces to a greater extent for the first time during the restricted period or traveling to more remote locations in order to spend time in green space with fewer people^26,27^. In essence, many individuals were seeking to be in outdoors spaces in a socially distanced manner; however, the type of setting and location of space may have differed based on social, emotional, and physical needs.

In this research paper, we examine how individuals in two cities in Australia chose to spend time in green space during the restricted period of the Covid-19 pandemic and what impact difference in public green space versus private yard use had on their mental health—specifically on depression, stress, and anxiety as well as their personal wellbeing. Given limited amounts of time, some people may accrue their time in nature by visiting public green space while others may visit private yards more, but the amount of time in each setting may lead to different health profiles and benefits accrued. Depending on the context of the lockdowns, individual risk perceptions, and the context of the green spaces available there may have been some spaces that were considered more controllable or predictable to individuals. One hypothesis is that while opportunities to recreate, socialise, and exercise may exist in both public spaces and private yards, private yard spaces may be perceived as nature spaces where proximity to others or social encounters can be more controlled, and thus used as an alternative to restricted access public green spaces during the first year of the pandemic.

However, changes in urban landform may also impact on people’s ability to access public green spaces or private yards. Urban densification has led to public urban green space developed for other uses (e.g. residential or commercial development, easements for transportation networks), with the distribution of public green space often unevenly distributed in cities leading to social and economic inequality of access^30,31^. Such studies have been highlighted globally with disparities in urban green space accessibility significant between the richest and poorest census tracts as well as between racial or ethnic groups^31–33^. Shifting demographics also show that low-income groups have been transitioning from areas with more green space to areas with less green space over time^34^. This pattern of development points to the social injustice of green space access and the resulting health and wellbeing benefits that come with it^35^. Urban densification also impacts private green space availability, with infill development of suburbs leading to larger houses being built on smaller lots and a loss of private green space^36,37^. Often, no new public green space is provided to offset the loss^38^. Thus, it becomes increasingly important to understand how these two spaces, public green space and private yards, differ in the mental health and wellbeing benefits they provide to urban communities, especially when considering the restricted access to many public green spaces during this time and the inequalities of accessing an alternative private space during a stressful period.

**Fig. 1:**
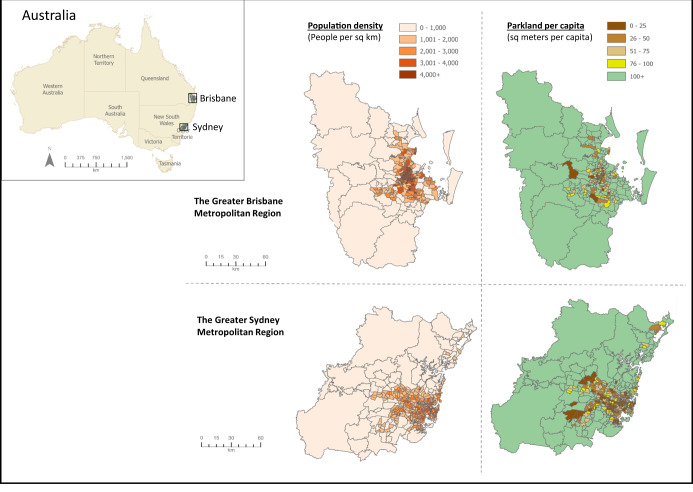
Maps of the Greater Brisbane Metropolitan Region and Greater Sydney Metropolitan Region—as defined by the Australian Bureau of Statistics. Maps are based on the same scale to show the relative size of each Metropolitan Region (Data by Region, https://dbr.abs.gov.au/). Population density and parkland per capita information is shown at the statistical area 2 level from the Australian Bureau of Statistics and represents a community that interacts together socially and economically. These maps show the relatively equal sizing of the metropolitan areas and the distribution of the population and park availability across the region.

In this study, we explore the differences in the role of public green space versus private yards for mental health and wellbeing during the first year of Covid-19, when restrictions were at its strictest and communities were highly uncertain about ramification to the exposure to Covid-19. We deployed an online green space and lifestyle survey (April 2021, *n* = 2084) to examine how urban dwellers were using public (e.g. parks, forests, beaches) as well as private (i.e. yards) green spaces in two cities in Australia, Brisbane (Queensland) and Sydney (New South Wales) (Fig. 1). Both cities are located on the east coast of Australia, located ~900 km apart. Sydney is the capital of New South Wales with a population of ~5.35 million over an area of 12,000 square kilometres^39^. Brisbane is the capital of Queensland with a population of ~2.6 million over an area of about 16,000 square kilometres^40^. Although Sydney is significantly bigger than Brisbane, both Sydney and Brisbane have experienced rapid growth over the last two decades, resulting in urban consolidation and a loss of public and private green space^39,41,42^. Sydney and Brisbane also both experienced a multi-month lockdown in the early months of Covid-19, which led to more sporadic lockdowns when Covid-19 was detected within the community.

The survey was deployed about 1 year after the initial lockdowns that began in mid-March 2020 and provided an opportunity to examine how the patterns of public green space and yard use related to differences in mental health and wellbeing scores over the preceding year. We asked respondents how often they visited public green spaces and yards over the year (frequency) and how much time they spent in public green spaces and yard last week (duration). It is important to note that these two measurements of green space (i.e. frequency and duration) use represent different aspects of green space visitation in terms of timing and intensity of use, but both are frequently used together to understand green space use behaviour^43,44^. While frequency often represents a more general pattern of use (e.g. how often a person visits), duration, in this study, focuses on how long a person spends in the type of green space. In the case of this study, frequency over a year was used to establish a general pattern of use; however, duration was considered only within the week prior to the survey in order to increase the accuracy of response (and in keeping with previous research on self-reported behaviour^45^) and represents a short and recent period of use.

To understand how different combinations of public green space versus yard visits are linked with mental health, the respondents were split into four different use categories based on the frequency and duration of use of public green space versus yard use. The respondents were stratified into the four quadrants based on the average scores as cut-off. For the frequency of nature experience, the respondents were stratified into the following four quadrants: high frequency in both private and public green space visits (f-HH), high frequency in private but low frequency in public green space visits (f-HL), low frequency in private but high frequency in public green space visits (f-LH), and low frequency in both private and public green space visits (f-LL). The same stratification was performed regarding the duration of time spent on nature experiences last week (d-HH; d-HL; d-LH; d-LL). Table 1 summarised the number of respondents in each quadrant and the cut-offs used to distinguish the quadrants. Using these categories, we examined how different combinations of uses may afford individuals with varying levels of health and wellbeing benefits as well as how the difference in measurements such as general frequency of use versus duration of use over a distinct, recent period of time may provide a different understanding of relationships between green space/yard use and mental health and wellbeing based on timeframe of use.

**Table 1:** Number of respondents in each of four quadrants of public and private green space use for frequency (f-) and duration (d-) of nature experiences.

Frequency	High public green space use	Low public green space use	Duration	High public green space use	Low public green space use
High private yard use	814 (f-HH)	380 (f-HL)	High private yard use	459 (d-HH)	648 (d-HL)
Low private yard use	429 (f-LH)	461 (f-LL)	Low private yard	249 (d-LH)	719 (d-LL)

Frequency of use measures represent a general pattern of use over the year. Duration of use represents a distinct, recent period of use over the last week. The average was used as a cut-off to categorise respondents into high or low categories. On average, respondents visited yards once a week to 2–3 days a week (average score = 3.5) with between 31 min to 1 h and 1–3 h last week (average score = 2.7). Respondents visited public green spaces almost once a week (average score = 4.89) over the year with about 3 h last week (average score = 3.1).

## Results

### Mental health and wellbeing relationships to general frequency of use

**Fig. 2:**
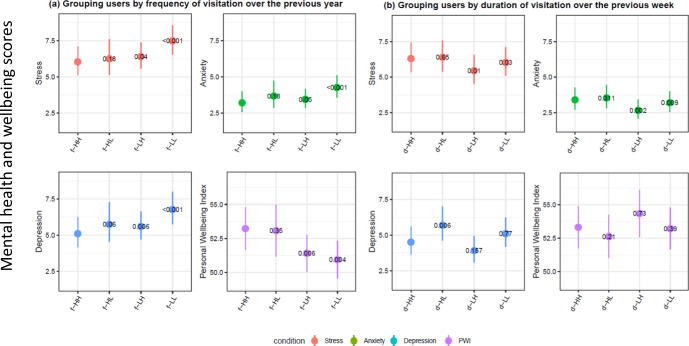
Examining differences in the four stratified end user types. The two panels represent differences across end user types based on frequency over the year (f-xx, panel **a**) and duration over the last week (d-xx, panel **b**) measures. The first x position represents yard use and the second x position represents public green space use (HH – high yard/high GS; HL – high yard/low GS; LH – low yard/high GS; LL – low yard/low GS; GS = public green space). Self-reported stress (orange), anxiety (green), depression (teal), and wellbeing scores (purple) are presented across the two different measure of use, frequency of use over the past year and duration of use in the week prior to the survey. The *y*-axis represents the average score for stress, anxiety, depression, and PWI (Personal Wellbeing Index), and figures include the estimate and 95% confidence intervals of the relationship between different combination of green space visits are presented after controlling for demographic factors.

We find that individuals in the group of low frequency users in both yard visits and green space visits over the previous year (f-LL) had the highest level of stress, anxiety, and depression and lowest personal wellbeing score across four quadrants (Fig. 2a). Individuals with the high frequency in both yard and green space visits over the previous year (f-HH) had the best mental health and personal wellbeing scores (Fig. 2a). Interestingly, compared to individuals with the high frequency in both yard and green space visits (f-HH), individuals in the group of low frequency in yard visits but high frequency in public green space visits (f-LH) had worse mental health and personal wellbeing; however, individuals in the group of high frequency in yard visits and low frequency in public green space visits (f-HL) did not differ from individuals with high frequency in both yard visits and public green space visits (f-HH) (Fig. 2a). This suggests that over the first year of the Covid-19 pandemic, a high frequency in yard visits (f-Hx quadrants, x representing the green space visitation) was associated with better mental health scores more strongly than frequency of green space visits.

### Mental health and wellbeing relationships to 1 week duration of use

In examining the results for duration of visit in the week prior to the survey, the results revealed a different pattern. Individuals in the groups of low duration use in yard visits (d-LL and d-LH) in the prior week reported having lower stress and anxiety than individuals with high duration in yard visits in the prior week (d-HH and d-HL), regardless of the duration in public green space visits (Fig. 2b). Individuals in the high yard and low public green space duration (d-HL) category exhibited significantly higher depression scores than the other categories of users (Fig. 2b).

### Additional result patterns

In addition, both age and income were negatively correlated with stress, anxiety, and depression and positively correlated with personal wellbeing scores (Table 2). Individuals with higher NR-Experience scores also reported higher wellbeing scores (Table 2). Individuals from Sydney reported higher levels of stress, anxiety, and depression (Table 2).

**Table 2:** Model results examining the relationship between different type of green space (GS) users and stress, anxiety, depression, and wellbeing adjusted for socio-demographic factors.

STRESS	est	SE	*p*	ANXIETY	est	SE	*p*
(Intercept)	2.790	0.179	<0.001		2.887	0.234	<0.001
Freq (f-HL)	0.099	0.074	0.181		0.134	0.099	0.177
Freq (f-LH)	0.163	0.079	0.039		0.209	0.107	0.050
Freq (f-LL)	0.303	0.076	<0.001		0.456	0.098	<0.001
Duration (d-HL)	0.032	0.070	0.645		0.094	0.092	0.311
Duration (d-LH)	−0.247	0.097	0.011		−0.400	0.132	0.002
Duration (d-LL)	−0.174	0.079	0.029		−0.274	0.105	0.009
NRExp	0.014	0.036	0.704		−0.047	0.048	0.325
Age	−0.104	0.008	<0.001		−0.148	0.010	<0.001
Gender (male)	−0.075	0.049	0.131		−0.020	0.065	0.754
Income	−0.018	0.009	0.052		−0.037	0.012	0.002
City (Sydney)	0.164	0.049	0.001		0.359	0.066	<0.001
Education	−0.008	0.010	0.451		−0.022	0.013	0.094
DEPRESSION	est	SE	*p*	PWI	est	SE	*p*
(Intercept)	2.822	0.222	<0.001		38.086	1.859	<0.001
Freq (f-HL)	0.174	0.092	0.058		−0.692	0.739	0.349
Freq (f-LH)	0.271	0.098	0.006		−2.157	0.786	0.006
Freq (f-LL)	0.384	0.094	<0.001		−2.311	0.796	0.004
Duration (d-HL)	0.251	0.090	0.006		−0.899	0.709	0.205
Duration (d-LH)	−0.178	0.126	0.157		−0.337	0.961	0.726
Duration (d-LL)	−0.030	0.102	0.770		−0.700	0.805	0.385
NRExp	−0.023	0.044	0.601		1.720	0.359	<0.001
Age	−0.101	0.009	<0.001		0.500	0.077	<0.001
Gender (male)	−0.028	0.061	0.652		−0.135	0.504	0.789
Income	−0.057	0.011	<0.001		0.670	0.097	<0.001
City (Sydney)	0.213	0.061	<0.001		−0.806	0.498	0.106
Education	−0.014	0.012	0.259		0.147	0.101	0.147

We ran three generalised linear models for stress, anxiety, and depression with quasi-Poisson error structure to account for overdispersion with stress, anxiety, and depression as the response variables for each of the models. We ran a linear regression model for personal wellbeing index, and the explanatory variables and covariates are the same as those in the generalised linear models.

Freq = frequency of green spaces visits over a year. Duration = duration of green space visits last week.

*HH* high yard/high GS, *HL* high yard/low GS, *LH* low yard/high GS, *LL* low yard/low GS use, *Est* estimate, *SE* standard error, *p* p value.

To test the robustness of the result, an additional analysis was run without using the four quadrants in frequency and those in duration, we used separate variables for yard and green space visits with interaction terms. The results are consistent (Supplementary Table 1). In short, individuals visited yard less often than the average tended to report having better mental health; in contrast, individuals spent less time in visited yard last week than the population average tended to report having better mental health (Supplementary Table 1). Public green space visits in duration or frequency did not have significant association with mental health, except for depression.

## Discussion

The research examines the role of both public and private green spaces in providing urban residents mental health and wellbeing benefits based on patterns of use and socio-demographic traits. When examining the frequency of visits (general pattern over the year) to these two types of green space over the first year of Covid-19 restrictions, we found that individuals who visited their yard more often than the population average reported having better mental health and wellbeing. Such results confirm previous research that spending time in green space can confer mental health and wellbeing benefits resulting in lower stress, anxiety, and depression scores, while supporting high levels of personal wellbeing^46,47^. However, the reverse is observed when examining duration of visit over the prior week with more time spent in the yard showing patterns of increasing stress, anxiety, and depression compared to those with less than average use. Such a result highlights a thought-provoking pattern on why frequency and duration results do not have the same directional effect.

While other studies have shown that duration and frequency are primarily correlational and that increased nature contact improves wellbeing benefits, in many of these studies, the frequency and duration categories are connected variables —for example, asking respondents to self-report how frequently they spend time in nature and the average length of duration for their visits^48,49^. Thus, both measures are asking for a general pattern of use in green spaces. In these studies, the results show a positive correlation between the reported frequency and average duration of visits in relation to health benefits. Because self-reported measures are prone to errors related to retrospective memory^48^, we have chosen to ask respondents to report on the durations of time spent in public green space or yards in the past week, a time period that allows for greater accuracy in recall^47^. However, this is also a different measure than what has been used in other research which asked respondents to provide an average duration of time per visit over a longer time period.

Because of this difference, the results are not counterintuitive, as they first might seem. They provide new information into research on nature dose and health and wellbeing benefits because it presents a different type of dose relationship to examine. Although we cannot determined from the data collected the origin of high self-reported stress, anxiety, and depression in high duration yard users (d-HH, d-HL), this prompts us to consider why high duration use of yard in the immediate past (1 week) would be correlated to lower mental health scores. It is important to remember that correlations do not translate into causation as there may be other factors or contexts that are influencing the duration that individuals spend in their yards; however, one conjecture may be that respondents sought to spend more time in their yard in that time period for the express purposes of emotional regulation because they were suffering from stress, anxiety, or depression around the time of the survey collection. In response to their immediate situation, such individuals may have sought greater time in green space, and potentially in a very close-by green space, their yard.

The desire to spend time in natural areas to improve mental health has been shown in the literature. A study across 18-countries showed that people with common mental health disorders often use nature to help regulate their emotions; however, social processes such as perceived social pressure to visit nature can also trigger anxiety responses in individuals^50^. In addition, a study in China examined the behaviour of individuals who considered themselves to be stressed and found that the highest stressed respondents sought out serene nature spaces for stress recovery^51^. Thus, highly stressed individuals may seek to spend more time in green space in a particular week to alleviate some of the mental health load. A meta-analysis on nature exposure on mood also found that nature contact impacts positive emotions more that negative emotions, meaning that while there are improvements in positive mood, there is less of an impact on negative emotions, but short visits of even 5 min can provide some respite from mental health pressures^48,52^. Ongoing research is attempting to understand how stress, anxiety, and depression influences time spent in nature and how the ‘dose’ of nature impacts on positive and negative emotions.

It is also important to note that this survey was deployed about a year after the Covid-19 restrictions began, and changes in behaviour in terms of social isolation, loss of employment, concerns for older family members, and more impacted mental health and wellbeing of the respondents as week as their decision-making around which green space to use, how often and for how long. Research has also found that individuals who spend more time at home report more severe symptoms of depression^53^, and reasons such as disability, chronic pain, and fatigue may make it difficult to leave the house, leading to reduced mobility out of the home and limited community participation^54^. While the results are unable to provide a clear understanding of causation, they highlight that more research is required to understand the differences between general frequency and duration measures versus frequency and duration measures over short and specific periods of time especially depending on both the context of the space (e.g. a park versus a yard) as well as understanding the personal circumstances that are driving these decisions around use of green space. A longer or more frequent set of visits over a short period of time may signal an immediate response to poor mental health; however frequent use of longer visits may provide a long-term pathway toward better mental health and wellbeing. Future studies to disentangle these patterns may require cohort designs or longitudinal studies that allow for the ability to study changing contexts through time to better understand how changing circumstances affect individual behaviour regarding green space use and further elucidate the different roles the public green space may play from yards.

Certain socio-demographic variables were also significant predictors for mental health and wellbeing. Older and high-income individuals reported lower stress, anxiety, and depression while reporting higher wellbeing scores. During Covid-19, these individuals were more established with jobs that could be performed at home, suffered less job loss, had more savings, and lived in homes that were larger and with yards to allow for more comfortable isolation periods^55–57^. We also found that respondents in Sydney reported significantly more stress, anxiety, and depression than respondents from Brisbane. While the data collected from the survey are unable to clearly delineate the reasons behind this, urban form and structure is more compact in Sydney than Brisbane leading to less spacious and more expensive housing and Covid-19 pressures led to more restrictive patterns of lockdown. Previous work in Sydney has shown that neighbourhood dwelling density is associated with reduced public and private green space, although suburbs of higher socio-economic advantage had significantly more private green cover, and disadvantaged communities rely on public green^58^. This finding is consistent with work in other cities where both private and public green space decreases as urban density increases and land use intensifies^59^. Brisbane, on the other hand, has experienced continued growth through Covid-19, most likely due to individuals moving from larger cities in search of bigger house, more green space, and more freedoms from lockdowns^60,61^. Greater social research to understand the factors leading to differences in stress, anxiety, and depression are warranted to consider why self-reported mental health scores are lower in Sydney.

In our study, additional to providing evidence supporting the importance of green space for mental health and wellbeing, we also highlight the importance of contexts, such as types of green space, existing condition of mental health, and different measurement of nature experiences, and requirements of individual are necessary to gain a clearer understanding of the drivers of green space use, whether is it private or public space. For instance, private and public green spaces often have different functions and may not substitute for each other in the way they are used^62^. Private residential green spaces provide individuals a self-determine area where there is greater control over the environment and social interactions, while public green spaces require end users to assess each space for their requirements specific requirements at that time in terms of type of space, management, and other people and their use of the space.

As green spaces in cities are at a premium for mental health and well-being benefits, providing easily accessible and readily available green space is necessary for our rapidly growing urban populations. Green space, and especially private residential green spaces, are becoming increasingly unavailable, yet a desire for ‘safe’ green spaces were highly desired during the restricted periods of the Covid-19 pandemic. With the potential for new pandemics in the future, an important policy question is how to provide safe spaces for the greater urban community. Debates around the distribution of public versus private land are ensconced in issues of equity and policy, especially considering the significant benefits that are afforded to urban residents who can and do access green space^63^ and for the residents who depend on them for their mental health^51^. For those with less access to private residential green spaces, solution sets must be developed to allow for social distancing and proximity control, while still providing the functions and activities that people desire. Ideas of temporal zonation, as was done in many grocery stores during the pandemic^64^, could be implemented into park programming. New designs of spaces that allow for socially distanced community interactions can be tested with communities to ensure that the green spaces meet their needs. Such learnings will have important implications for how to ensure these safe green spaces are available to everyone will shed light on how urban planning and development policies can increase and create equity in wellbeing benefits.

## Methods

### Survey information

An online survey was conducted between April 15th and May 15th 2021 for Brisbane and Sydney residents asking about nature experiences within their city. This research was conducted in accordance with approved guidelines, and all protocols were received under Institutional Human Research Ethics Approval (CSIRO Human Research Ethics Review Board, Project 144/20). Informed consent was obtained from all respondents prior to starting the survey.

The survey was delivered by an online data collection company, the Online Research Unit (https://www.theoru.com/index.htm), a general market and data analysis company well-established in Australia, to run a survey panel through their existing research databases of potential respondents in each city. The time period was chosen as it was during a time when seasonal temperatures would not affect participation in going to nature spaces. The company maintains a large database of individuals within both cities from which to sample. In order to obtain a demographically representative sample, demographic parameters were provided to the company to ensure that sampling occurred across gender, education, and income variable to be representative of each city. A minimum high quality sampling number was requested from the survey company (*n* = 1000) based on the demographic parameters. Discussions with company representative statisticians with the project team were conducted to determine that a sample of *n* = 1000 would provide enough respondents in each category across demographic variables for sufficient power in statistical analyses. A total of 1050 respondents were captured from Brisbane, and 1034 surveys were collected from Sydney for a total 2084 responses.

### Socio-demographic information and nature connection

For this study, a large range of questions were asked regarding a survey participant’s self-reported socio-demographic information, their use of green space, nature connection, mental health and personal well-being. Socio-demographic questions included information on age, gender, education level, and income level. Survey participants were asked to complete the Nature Relatedness Scale (referred to as ‘NR’ here) to assess their level of nature connection^65^. This scale requires participants to complete a series of questions that assess the affective, cognitive, and experiential relationship individuals have with the natural world across 21 statements. These responses were then scored and calculated according to the process presented in Nisbet et al. ^65^. A higher average score indicates a stronger connection with nature. The scale has been demonstrated and validated to differentiate between known groups of nature enthusiasts and those not active in nature activities, as well as those who do and do not self-identify as environmentalists. It also correlates with environmental attitudes and self-reported behaviour and appears to be relatively stable over time and across situations^65^. Because we are specifically asking about the use of green space by individuals, we isolate this variable to the 6 statements which specifically ask about an individual’s associations with nature through their physical relationship and familiarity of nature—which is called ‘NR-Experience’^65^. The ‘NR-Experience’ measure has also been validated through previous research^65^.

#### Green space visitation—in public green space and private yards

We asked respondents to assess both their frequency of visits to public and private green space as well as the estimate a typical amount of time that they spend in either space in a week to assess the duration of time spent in either type of green space.

*For public green space*: Respondents were asked about their general pattern of visitation to parks and other outdoor public nature areas over the last year to understand an individual’s general frequency of use. These include both urban and peri-urban spaces with examples provided in the question, as can be seen below. Respondents were also asked about the public green spaces they visited in the last week and the total amount of time spent in those green spaces. We chose the timeframe of a week to ask about duration of time spend because it provided a short and recent reference period to improve accuracy^45^. This amount was self-reported based on the following categories:

- *Frequency of public green space visits:* Participants were asked to recall about how often they usually visit or pass through outdoor green spaces for any reasons. The frequency was selected from the following categories: never (=0), once a year (=1), once every 3 months (=2), once a month (=3), 2–3 times a month (=4), once a week (=5), 2–3 days a week (=6), 3–5 days a week (=7), and 6–7 days a week (=8).
- *Duration of public green space visits*: Participants were asked to recall over the last week what outdoor green spaces they visited or travel through and to estimate the total time they spent there in hours. Participants spent more than 10 h in a week in public green spaces were coded as 10. This includes, for example, beaches, children’s playgrounds, parks, bushland, bike‐ways, picnic areas, beaches, golf courses, tennis courts and bowling greens. Participants who reported spending more than 168 h or <0 h in public green spaces were considered as error (“NA”). Participants who did not visit any public green spaces were considered as zero duration of public green space visits last week.

*For private green spaces*: Respondents were asked about their general pattern of use of private yards and gardens over the last year to understand an individual’s general frequency of use of private green space. This amount was self-reported based on how often they spent more than 10 min in their yard. Respondents were also asked how much time they spent in private yards and gardens over the last week to gain a sense of the duration of usage over a week. Again, we chose this timeframe as it provided a short and recent reference period to improve accuracy^45^. Our definition does not include other forms of private green space such as those under the jurisdiction of schools, corporations, aged care facilities or religious institutions.

- *Frequency of yard visits:* Participants were asked to recall how often they usually spend more than 10 min in their own yard or on their deck. The frequency was selected from the following categories: I don’t have a yard or deck (=0), never (=0), less than once a month (=1), 2–3 times a month (=2), once a week (=3), 2–3 days a week (=4), 4–5 days a week (=5), and 6–7 days a week (=6). Participants chose “I don’t have a yard or deck) were considered as zero frequency in yard visit. People who did not have a yard were included in the analysis as coded as 0 because they still represented a set of people who had had 0 or no experience with a private yard when considering the analysis based on “nature experience” and the NR-experience subscale used.
- *Duration of yard visits last week*: Participants were also asked to think about the last week, how much time in total they spent in their own yard or on their deck. The duration was selected from the following categories: No time (0), 1–30 min (1), 31 min to 1 h (2), 1–3 h (3), 3–5 h (4), 5–7 h (5), 7–9 h (6), more than 9 h (7). Similar to the frequency of yard visits, participants chose “I don’t have a yard or deck) were considered as zero duration of yard visit last week.

#### Mental health and well-being measures

Mental health and well-being were assessed based on two commonly used measures.

Mental health measures were conducted using the commonly used Depression, Anxiety and Stress scale (DASS-21) self-reported questionnaire consisting of 21 items, with 7 items per subscale (to measure an individual’s self-assessment of their depression, anxiety, and stress status)^66^. Patients were asked to score every item on a scale from 0 (did not apply to me at all) to 3 (applied to me very much). Sum scores were computed by adding up the scores on the items per (sub)scale and multiplying them by a factor 2 and indicate indicates mild or worse depression. The DASS-21^66^ has been shown to be a valid and reliable measure of the dimensions of depression, anxiety, and stress with high internal consistency across a variety of settings^67^. The measures of depression, anxiety, and stress are general measure of mental health used in the public health and medical sector, and they are not based on a specific context or situation. The DASS has been used and tested in various Australian surveys such as the Australian Psychological Society Stress and well-being survey of Australia in 2015^68^, the Australian Research Council (ARC) Centre of Excellence for Children and Families Over the Lifecourse survey^69^, and in an assessment of measures of mental health in Australia during Covid-19^70^.

Personal wellbeing was assessed using the Personal Wellbeing Index developed by the Australian Centre on Quality of Life (ACQL, www.acqol.com.au). This consortium research group examines quality life as both an objective and subjective dimension, which comprises of several domains. These domains together define the total construct^71^. This scale contains seven items of satisfaction, each one corresponding to a specific quality of life domain which includes: standard of living, health, achieving in life, relationships, safety, community-connectedness, and future security. The PWI has been validated across user groups and is used in cross-cultural settings^72^.

### Analysis

All analyses outlined here were conducted in the software package R v4.2.2^73^.

We stratified participants into four quadrants for frequency of nature experience (frequency) and duration of nature experience (duration). For the frequency of nature experience, using the average as a cut off, we stratified participants as four quadrants: high frequency in both yard and green space visits (f-HH), high frequency in yard visits but low frequency in green space visits (f-HL), low frequency in yard visits but high frequency in green space visits (f-LH), and low frequency in both yard and green space visits (f-LL). We did the same to stratified participants based on their duration of nature experiences(d-HH; d-HL; d-LH; d-LL). See Table 1 for the number of participants in each group.

We then ran three generalised linear models for stress, anxiety, and depression with quasi-Poisson error structure to account for overdispersion. Stress, anxiety, and depression were the response variables for each of the models. Explanatory variables were the frequency of nature experience (f-HH as the baseline), duration of nature experience (d-HH as the baseline), desire to be in nature (NR-Experience subscale), and covariates are age, gender, income, city, and education. We ran a linear regression model for personal wellbeing index, and the explanatory variables and covariates are the same as generalised linear models. The assumptions of model homoscedasticity and normality were fulfilled. Because the information of frequency and duration of visits are based on different time scales and with different types of responses, they represent different information about how individuals use and spend time in these green spaces. We also did not detect multicollinearity (VIF < 3) among explanatory variables.

To test the robustness of the results, we also ran additional analyses without stratifying participants into four quadrants. We ran identical models as previously described except that, instead of using the four quadrants in frequency and those in duration, we used separate variables for yard and green spaces visits with interaction terms. Specifically, we used high versus low frequency in yard visits (f-Hx; f-Lx), high versus low frequency in green space visits (f-xH; f-xL), high versus low duration in yard visits last week (d-Hx; d-Lx), and high versus low duration in green space visit last week (d-xH; d-xL). We also included the interaction term between the two frequency comparisons and between the two duration comparisons in the model. The results are consistent with the above analysis (Supplementary Table 1).

## Supplementary information

Supplementary Information

## Data Availability

The datasets aggregated and/or analysed during the current study are available from the corresponding author on reasonable request and will be made available on an online repository at a later date.
