# Supplementary material for: Nature experience from yards provide an important space for mental health during Covid-19
Source: NPJ Urban Sustain. 2023 Mar 10;3(1):14. doi: 10.1038/s42949-023-00094-0 (PMC9999340; doi:10.1038/s42949-023-00094-0)
Supplement: Supplementary file 1 — Supplementary Information [file 42949_2023_94_MOESM1_ESM.pdf]

Supplementary Information

Nature experience from yards provide an important space for mental health during Covid-19

Brenda B. Lin, Chia-chen Chang, Thomas Astell-Burt, Xiaoqi Feng, John Gardner, and Erik Andersson

Supplementary Table 1. Model results examining stress, anxiety, depression, and PWI (Personal wellbeing index) based on sociodemographic data and green space use data. These sets of models did not stratify survey respondents into four quadrants of frequency or duration urban green space use (GS). Instead, separate variables for private and public green space use were used (low: lower than population average, high is used as a baseline) within the model with interaction terms as additional terms. Because the respondents are not stratified – we have used the term 'x' to account for the fact that there is no low or high category for the opposite type of space (yard or GS). As in the primary presented analysis, we ran three generalized linear models for stress, anxiety, and depression with quasi-Poisson error structure to account for overdispersion with stress, anxiety, and depression as the response variables for each of the models. We ran a linear regression model for personal wellbeing index, and the explanatory variables and covariates are the same as those in generalized linear models. Freq = frequency of green spaces visits over a year. Duration = duration of green space visits last week. Hx – high yard use; xH – high GS; Lx – low yard use; xL –low GS. Est=estimate, SE = standard error, p=p-value.

	Stress				Anxiety				Depression				PWI		
	est	SE	p		est	SE	p		est	SE	p		est	SE	p
(Intercept)	2.790	0.179	0.000		2.887	0.234	0.000		2.822	0.222	0.000		38.086	1.859	0.000
Freq Yard Low (f-Lx)	**0.163**	**0.079**	**0.039**		**0.209**	**0.107**	**0.050**		**0.271**	**0.098**	**0.006**		**-2.157**	**0.786**	**0.006**
Freq GS Low (f-xL)	0.099	0.074	0.181		0.134	0.099	0.177		0.174	0.092	0.058		-0.692	0.739	0.349
Duration Yard Low (d-Lx)	**-0.247**	**0.097**	**0.011**		**-0.400**	**0.132**	**0.002**		-0.178	0.126	0.157		-0.337	0.961	0.726
Duration GS Low (d-xL)	0.032	0.070	0.645		0.094	0.092	0.311		**0.251**	**0.090**	**0.006**		-0.899	0.709	0.205
NR-Exp	0.014	0.036	0.704		-0.047	0.048	0.325		-0.023	0.044	0.601		**1.720**	**0.359**	**0.000**
Age	**-0.104**	**0.008**	**0.000**		**-0.148**	**0.010**	**0.000**		**-0.101**	**0.009**	**0.000**		**0.500**	**0.077**	**0.000**
Gender(male)	-0.075	0.049	0.131		-0.020	0.065	0.754		-0.028	0.061	0.652		-0.135	0.504	0.789
Income	-0.018	0.009	0.052		**-0.037**	**0.012**	**0.002**		**-0.057**	**0.011**	**0.000**		**0.670**	**0.097**	**0.000**
City (Sydney)	**0.164**	**0.049**	**0.001**		**0.359**	**0.066**	**0.000**		**0.213**	**0.061**	**0.000**		-0.806	0.498	0.106
Education	-0.008	0.010	0.451		-0.022	0.013	0.094		-0.014	0.012	0.259		0.147	0.101	0.147
Freq Yard Low (f-Lx): Freq Public GS Low (f-xL)	0.040	0.101	0.690		0.113	0.134	0.399		-0.061	0.124	0.621		0.538	1.044	0.606
Duration Yard Low (d-Lx): Duration Public GS Low (d-xL)	0.041	0.108	0.705		0.033	0.146	0.822		-0.103	0.137	0.453		0.536	1.080	0.620
